# Paroxysmal nocturnal hemoglobinuria in a patient post COVID‐19 virus infection: A case report with literature review

**DOI:** 10.1002/ccr3.8011

**Published:** 2023-10-09

**Authors:** Mohammed Albalawi, Turki Alwasaidi, Mohammed Almohammadi, Ashraf Ahmad Alzarief, Ghaleb Elyamany

**Affiliations:** ^1^ Department of Internal Medicine, College of Medicine Taibah University Madinah Saudi Arabia; ^2^ Medicine Department Prince Mohammed Bin Abdul‐Aziz Hospital‐National Guard Hospital Madinah Saudi Arabia; ^3^ King Abdullah International Medical Research Center King Saud bin Abdulaziz University for Health Sciences, Pathology and Laboratory Medicine, King Abdulaziz Medical City Jeddah Saudi Arabia; ^4^ Department of Neurosurgery Cairo University Cairo Egypt; ^5^ Central Military Laboratory and Blood Bank Prince Sultan Military Medical City Riyadh Saudi Arabia

**Keywords:** Complements, COVID‐19, PNH

## Abstract

COVID 19 is a serious infection that originated in Wuhan, China and has resulted in worldwide morbidity and mortality. It continues to be a major health concern in 2022, being associated with multiorgan failure. Although the pathophysiology of the disease and its complications are not well understood, it is believed that a cytokine storm, triggered by complement activation may be responsible for the severity and complications of the disease. As of now, there is no definitive treatment available. Hematological changes associated with COVID‐19 include lymphopenia, anemia, thrombocytopenia, disseminated intravascular coagulopathy, and thrombosis. Paroxysmal nocturnal hemoglobinuria (PNH), on the other hand, is an acquired clonal hematopoietic stem cell disorder that occurs due to an acquired PIG‐A mutation affecting the hematopoietic stem cells. Interestingly, PNH exhibits some clinical and laboratory manifestations like those seen in COVID‐19. In this report, we present a rare case of PNH that developed following a COVID‐19 infection.

## Graphical abstract

Hypocellular bone marrow biopsy for the patient's age.

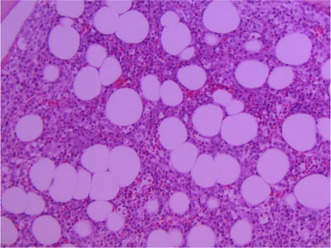

## 1 INTRODUCTION

Coronaviruses are a large family of zoonotic viruses that cause a range of illnesses from common flu‐like symptoms to severe respiratory disease. Several strains of coronaviruses have caused diseases in humans, including COVID‐19, severe acute respiratory syndrome (SARS‐CoV), and Middle East respiratory syndrome (MERS‐CoV).^1^, ^2^

The coronavirus outbreak, which began in December of 2019, originated in Wuhan, China. The virus is believed to have been transmitted from animals to humans. According to the World Health Organization (WHO), the main modes of transmission are through air‐droplets, aerosol, or direct contact between individuals. The incubation period of the virus which refers to the time from exposure to the onset of symptoms, typically ranges from 1 to 2 weeks.^1^, ^2^ The pathophysiology of COVID‐19 and its associated complications is still not clearly understood. Current publications suggest that complement activation plays a significant role in the pathogenesis of COVID‐19. The complement system functions to eliminate immune complexes, facilitate opsonization, induce cell lysis, and initiate an inflammatory process. The complement system consists of three main pathways: classical, alternative, and lectin pathways. Each pathway is stimulated through specific mechanisms and has proximal components (C1–C3) and terminal components (C5–C9).These pathways work collaboratively to produce the membrane attack complex (MAC) which includes complements C5–C9.^3^, ^4^ Complement pathways inhibition rely on two major proteins expressed on the cell membrane of various cells in the body: CD55 and CD59. CD55, previously known as decay accelerating factor (DAF), is a glycosylphosphatidylinositol (GPI) anchored membrane protein widely expressed on different cells. Its main role is to inhibit and accelerate the decay of classical and alternative pathways C3 convertase. CD59, also known as MAC inhibitor, is a GPI‐anchored membrane protein that acts on the terminal pathway components. It prevents the assembly of the MAC by inhibiting the insertion of C9 catalyzed by C5B‐8 into the lipid bilayer of the pathogen.^3^, ^4^

Paroxysmal nocturnal hemoglobinuria (PNH) is a rare, acquired syndrome with a 30% mortality rate within 5 years. PNH is an acquired clonal hematopoietic stem cell disorder caused by an acquired PIG‐A mutation that affects the hematopoietic stem cells. This mutation leads to affected blood cell lines that are deficient in glycosylphosphatidylinositol (GPI)‐anchored proteins, specifically CD55 and CD59, which are important complement regulatory proteins. PNH shares some similarities to COVID‐19 in terms of clinical and laboratory manifestations (Table 1).^5^, ^6^, ^7^

**TABLE 1:** Comparison of clinical and laboratory manifestation of PNH and COVID‐19.

Clinical finding	PNH	COVID‐19
Anemia	Yes	Yes
Thrombocytopenia	Yes	Yes
Lymphopenia	No	Yes
Thrombosis	Yes	Yes
Renal diseases	Yes	Yes
Respiratory diseases	Yes	Yes
Hemolysis	Yes	Not significant
DIC	No	Yes
Complement activation	Yes	Yes
Others (multiorgan failure)	No	Yes

## 2 CASE REPORT

A 28‐year‐old gentleman, previously in good health, presented 1 month after being diagnosed with COVID 19 infection. He experienced mild COVID‐19 symptoms but did not require any treatment as he was asymptomatic. However, a few weeks later, he was presented with severe abdominal pain, necessitating surgical exploration. The CT scan of his abdomen revealed evidence of splenic infarction. Subsequently, he underwent splenectomy, and examination of the spleen specimen confirmed infarction with no signs of malignancy. Due to his anemia and thrombocytopenia, he was referred to our hospital. The laboratory results upon his presentation to our hospital are provided in Table 2.

**TABLE 2:** Laboratory findings in our patient at presentation with PNH.

Test	Patient ‘s value	Normal value
WBC	10–20 × 10^9^/L	4–11 × 10^9^/L
HGB	8–10 g/L	13–15 g/L
MCV	80–90	76–96
MCH	30–34	28–34
Platelet	15 × 10^9^/L	150–400 × 10^9^/L
LDH	197–259	140–280 U/L
Reticulocyte count	1.2	0.5%–2.5%
Coombs test (direct & indirect)	Negative	Negative
Total bilirubin/direct	0.90/0.40	1.2 (0.3) mg/DL
PT/INR	1.3–2.2	1–1.2
aPTT	35–50	30–40 s
Fibrinogen	529	200–400 mc/dL(2–4 g/L)
d‐dimer	11.3	<0.50
Lupus anticoagulant	33.8	20–39 GPL units
Anticardiolipin	IgG = 3, IgM <2	IgG < 23 GPL units, IgM < 11 MPL units
Beta2 glycoprotein antibodies	IgG = 3, IgM <2	IgG <1.0, IgM 9. 4–20 U/mL
ANA	3.8	
Anti‐DNA	<9.8	
CRP	25.8	<10 mg/L
ESR	2–16	0–15 mm/h
Urea	1 5‐40	5–20 mg/dL
Creatinine	0.40–0.75	0.74–1.35 mg/dL
COVID‐19	Negative	Negative

**FIGURE 1:**
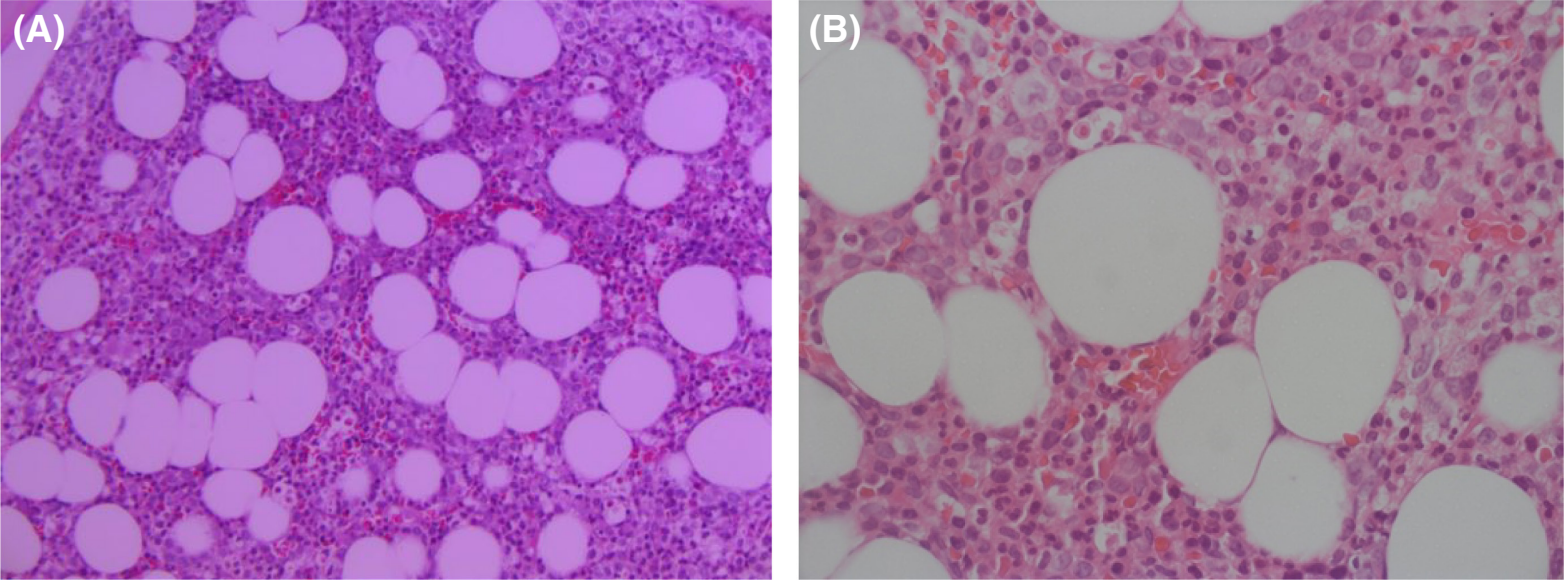
Low (A) and high power (B) microscopic image of bone marrow biopsy showing an overall hypocellular marrow for age (10× and 40×).

He had negative Coombs test, indicating non‐immune‐mediated anemia, and thrombocytopenia. Further investigations ruled out antiphospholipid syndrome and microangiopathic hemolytic anemia (MAHA), including TTP/HUS. His repeated COVID‐19 test came back negative. A bone marrow biopsy (Figure 1A,B) revealed mild hypocellularity for his age (40%–50%) and trilineage hematopoiesis with a left shift and decreased megakaryocytopoiesis. No lymphoid aggregates, granuloma or atypical infiltrate were observed.

**FIGURE 2:**
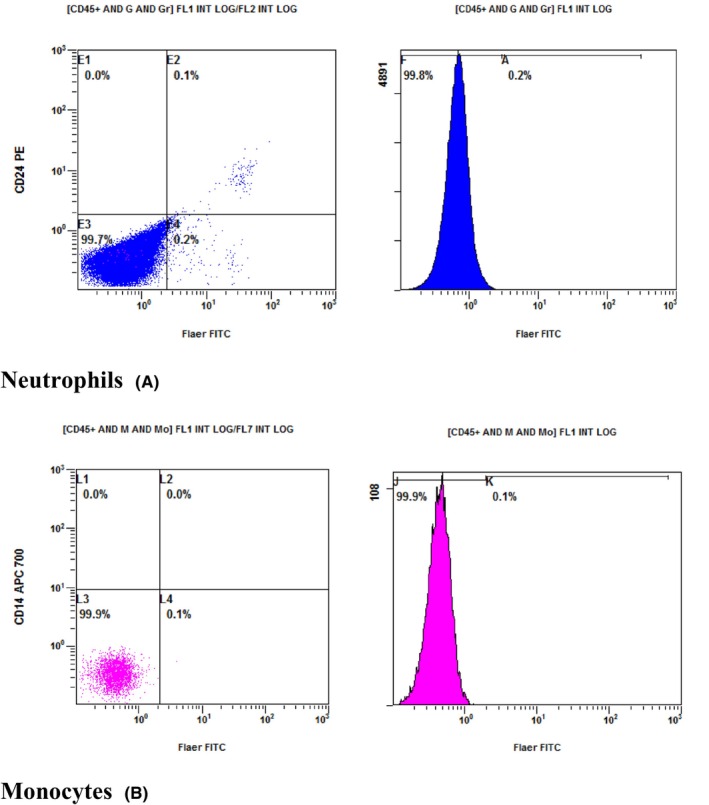
(A, B) Selected flowcytometry analysis plots showing the confirmation of loss of GPI anchored proteins from surface of WBCs using (FLAER).

Chromosomal analysis was not conducted as there was no specific indication for it. Peripheral blood flowcytometry was performed to investigate the presence of an underlying PNH clone. The results of the flow cytometry are presented in Table 3 and Figure 2A,B.

**TABLE 3:** Flowcytometry results that showed significant PNH clone.

Red blood cells	GPI deficient RBCS	Type III (complete CD59 deficiency): 0.3%
		TYPE II (partial CD59 deficiency): 0.1%
		Total RBCS PNH clone size (Type II and Type III): 0.4%
Granulocytes	GPI deficient granulocytes (FLAER/CD24 deficiency)	99.8%
Monocytes	GPI deficient monocytes (FLAER/CD14 deficiency)	99.9%

These findings are indicative of a diagnosis of PNH.

During his hospital stay, the patient experienced complications including intracerebral hemorrhage (ICH), which necessitated surgical evacuation. Unfortunately, eculizumab and other complement inhibitors were not accessible for treatment. Despite receiving intravenous immunoglobulin (IVIG), steroid and supportive blood products the patient's condition deteriorated, and he ultimately succumbed to a massive pulmonary embolism that resulted in cardiopulmonary arrest.

## 3 DISCUSSION

COVID 19 is recognized as a systemic disease with the potential to affect multiple systems, including the hematological system. Hematological changes commonly observed in COVID‐19 patients encompass anemia, lymphopenia, thrombocytopenia, and thrombosis. Elevated ferritin levels have been identified as a poor prognostic factor in COVID‐19 patients Unfortunately, these changes have often been reported in isolation without being placed within the appropriate context. Complement activation is considered a major contributor to the progression, severity and complications of COVID 19.Deposition of complement components has been documented in various organs, such as the lungs, kidneys, endothelium, and skin, in COVID‐19 patients.^5^

PNH is a rare condition that affects approximately 1.13 individuals persons per million in the general population. PNH is an acquired disorder caused by a mutation in the PIG‐glycosylphosphatidylinositol (GPI) anchor, affecting proteins like CD55 and CD59. These mutations can be triggered by various factors including viral infections, exposure to chemicals, or radiation, but in many cases the cause is idiopathic. The diagnosis of PNH relies on both clinical presentation and laboratory findings (Table 4).

**TABLE 4:** Classification of PNH.

Parameter	Classical PNH	PNH in association with BM failure syndromes (AA/MDS/MF)	Subclinical PNH
Hemolysis	Marked	Mild	No
Hemoglobinuria	Present	Absent	Absent
LDH	Markedly elevated	Minimal	Absent
BM	Normal BM	Evidence of BM failure	Evidence of BM failure
PNH clone	PNH clone >50%	PNH clone <10%	PNH clone <1%

Clinical presentation of PNH is variable and includes Coombs‐negative hemolytic anemia, thrombocytopenia, and thrombosis at unusual site. Patients may also exhibit symptoms and signs such as dyspnea, dysphagia, abdominal pain, erectile dysfunction, renal failure, and pulmonary hypertension. PNH is often associated with other hematologic diseases, particularly bone marrow failure syndromes like aplastic anemia and myelodysplastic syndromes.^8^

Laboratory finding in PNH include Coombs‐negative anemia, thrombocytopenia, elevated lactate dehydrogenase (LDH), indirect hyperbilirubinemia, and increased reticulocyte count. However, the current diagnostic criteria for PNH rely on flowcytometry analysis. A diagnosis of PNH requires the demonstration of at least two different GPI protein deficiencies within two different cells lines, such as granulocytes, monocytes, or erythrocytes through flow cytometry.^8^, ^9^

The laboratory diagnosis of PNH is based on the following three criteria, as recommended by the International PNH Interest Group (IPIG).^9^

1. Evidence of non‐immune hemolysis.
2. Flow cytometric analysis demonstrating a population of peripheral blood cells deficient in GPI‐anchored proteins. Confirmation requires the identification of at least two different GPI protein deficiencies within two different cell lines from granulocytes, monocytes or erythrocytes.
3. Bone marrow aspirate, biopsy, and cytogenetics to assess the presence or absence of an associated bone marrow syndrome, such as myelodysplastic syndrome or aplastic anemia.

Various treatments have been employed, including intravenous immunoglobulin and steroids, although their efficacy lacks sufficient evidence.^10^ The mainstay of PNH treatment involves the use of complement inhibitors, such as eculizumab or ravulizumab, and/or allogenic stem cells transplantation. Despite our patient not receiving these medications due to their high‐cost and unavailability at our hospital, we believe they could be the treatment of choice not for PNH but also for covid‐19 infection itself.

Several ongoing studies are currently investigating the use of such medications for treating COVID‐19 infection. Our case report, along with supporting evidence from publications on complement activation associated with COVID‐19 infection, suggests the potential use of complement inhibitors as a treatment for COVID‐19 infection. However, the availability, cost, safety, and efficacy of these medications pose limitations.

Currently, there are ongoing clinical trials evaluating different complement inhibitors as a potential treatment for patients with moderate to severe COVID‐19 infection. We have summarized the ongoing trials involving complement inhibitors in COVID‐19 infection in Table 5.^10^

**TABLE 5:** Ongoing complement inhibitor trials for COVID 19 patients.

Drug	Mechanism of action	Trial phase
Eculizumab	C5 Inhibitor	III (EXPANDED)
Ravulizumab	C5 Inhibitor	III
AMY 101	C3 Inhibitor	II
APL‐9	C3 Inhibitor	III
Zilucoplan	C5 Inhibitor	II
Conestat Alfa	C1 Inhibitor	II
IFX‐1	C5a blockage	II and III
Avdoralimab	IgG1‐κ anti‐C5aR1	II
Narsoplimab	MASP‐2 inhibitor	

## 4 CONCLUSION

Our case report presents a rare occurrence of PNH following COVID‐19 infection, which have been rarely reported in the literature. Considering the limited efficacy and curative potential of currently available treatments, it is worth exploring complement inhibitor medications as an alternative therapy for patients with COVID‐19 infection. Conducting large scale trials to assess the effectiveness of these medications is crucial, taking into consideration their cost and availability. Additionally, we recommend studying the incidence of COVID‐19‐related PNH in patients presenting with COVID‐19 infection and cytopenia, as this has significant implications for the treatment of such patients.

## AUTHOR CONTRIBUTIONS

**Mohammed Albalawi:** Conceptualization; data curation; formal analysis; supervision; writing – original draft; writing – review and editing. **Turki Alwasaidi:** Data curation; formal analysis; validation; writing – original draft; writing – review and editing. **Mohammed Almohammadi:** Data curation; formal analysis; investigation; writing – original draft; writing – review and editing. **Ashraf Ahmad Alzarief:** Data curation; formal analysis; investigation; writing – original draft; writing – review and editing. **Ghaleb Elyamany:** Data curation; formal analysis; investigation; methodology; writing – original draft; writing – review and editing.

## FUNDING INFORMATION

None, this study was performed as part of employment of the authors (Department of Internal Medicine, College of Medicine, Taibah University, Madinah, Saudi Arabia.

## CONFLICT OF INTEREST STATEMENT

The authors have no conflict of interest to declare.

## CONSENT

No written consent has been obtained from the patient as there is no patient identifiable data included in this case report/series.

## Data Availability

The data is available on request
